# Altered wakeful theta activity characterizes levodopa-induced dyskinesia in Parkinson’s disease

**DOI:** 10.1038/s41531-026-01320-z

**Published:** 2026-03-19

**Authors:** Luigi Fiorillo, Giovanni Lombardi, Nicolo La Porta, Lisa Arnaud, Marco Veneruso, Anna Castelnovo, Ilaria Bertaina, Claudio Staedler, Alain Kaelin-Lang, Salvatore Galati

**Affiliations:** 1 Neurology Department, Neurocenter of Southern Switzerland, Movement Disorder Research Group, Ente Ospedaliero Cantonale, Lugano, Switzerland; 2 Institute of Digital Technologies for Personalized Healthcare, University of Applied Sciences and Arts of Southern Switzerland, Lugano, Switzerland; 3 Faculty of Biomedical Sciences, Università Della Svizzera Italiana, Lugano, Switzerland; 4 Institute of Information Systems and Networking, University of Applied Sciences and Arts of Southern Switzerland, Lugano, Switzerland; 5 Faculty of Informatics, Università della Svizzera Italiana, Lugano, Switzerland; 6 Department of Neurosciences, Rehabilitation, Ophthalmology, Genetics, Maternal and Child Health (DINOGMI), University of Genoa, Genoa, Italy; 7 Sleep Medicine Unit, Neurocenter of Italian Switzerland, Ente Ospedaliero Cantonale, Lugano, Switzerland; 8 Department of Psychiatric Neurophysiology, University Hospital of Psychiatry, University of Bern, Bern, Switzerland; 9 Department of Neurology, Inselspital, Bern University Hospital, Bern, Switzerland

**Keywords:** Diseases, Neurology, Neuroscience

## Abstract

Slow-wave activity during sleep facilitates synaptic downscaling, while theta activity during wakefulness reflects synaptic upscaling, and both processes may be altered in levodopa-induced dyskinesia (LID) in Parkinson’s disease (PD). We compared actigraphy and high-density EEG in 12 healthy volunteers and three PD cohorts: early stage (EPD, *n* = 12), advanced non-dyskinetic (ADV, *n* = 13), and advanced dyskinetic (DYS, *n* = 11). Participants completed one week of actigraphy monitoring, followed by two resting-state EEG recordings conducted separately in the morning and evening. Wake-theta activity was analyzed using both linear and linear mixed-effects models, adjusted for age/sex, plus cluster-based non-parametric statistics, then related to clinical variables, and actigraphy-derived sleep metrics via partial correlations. Dyskinetic patients showed marked sleep disruption, elevated morning theta compared with controls (*p* = 0.006, d = 1.54) and EPD (*p* = 0.03, d = 0.85), along with a significantly reduced diurnal theta build-up compared with controls (*p* = 0.009, d = 1.57). EPD and ADV groups showed preserved diurnal increases. In dyskinetic patients, a higher levodopa equivalent daily dose (LEDD) was correlated with higher morning theta (*ρ* = 0.70, *p* = 0.023, pFDR=0.046) and smaller diurnal theta increases (*ρ* = −0.77, *p* = 0.009, pFDR=0.046). Relationships between theta and actigraphy-derived sleep metrics were weaker and inconsistent across groups. These findings suggest a dyskinesia-specific profile of impaired wake-related theta homeostasis, motivating longitudinal studies combining polysomnography and waking EEG.

## Introduction

Parkinson’s disease (PD) is a slowly progressive, highly debilitating disorder and the second most common neurodegenerative disease^1^. PD mainly affects the motor system, with bradykinesia, resting tremor, rigidity, and posture instability as the main symptoms^2^. Beyond motor impairments, PD patients frequently exhibit olfactory and autonomic disturbances, along with sleep disorders, which are often among the earliest manifestations of the disease^3^. Circadian rhythm alterations and nocturnal sleep disturbance affects are extremely common in PD, affecting up to 90% of patients^4,5^. L-dopa is the most effective therapy for treating PD. As the disease progresses, patients may develop unexpected or paradoxical responses to the medication. While L-dopa is effective in managing motor symptoms in PD, patients may eventually develop motor fluctuations and abnormal involuntary movements, known as L-dopa–induced dyskinesia (LID), regardless of the duration of treatment. This long-term complication can result in significant functional disability and may necessitate complex pharmacological adjustments or surgical interventions.

Although sleep disturbances are part of PD’s clinical presentation, it remains unclear how sleep directly affects disease progression and whether sleep manipulation could provide therapeutic benefits. Slow-wave sleep (SWS) and the related slow-wave activity (SWA) are significantly reduced in PD patients^6,7^. Many patients in advanced disease stages, particularly those experiencing motor fluctuations, report improved motor performance upon awakening, known as the sleep benefit^8,9^. The state of vigilance can significantly influence clinical PD features, including rigidity and basal ganglia neuronal activity^10^. Poor nighttime sleep is positively associated with LID^11^, further reinforcing the link between sleep, PD, and the dopaminergic network. Clinical fluctuations and dyskinesias remain a major clinical issue in the long-term management of therapy-related complications, and compelling evidence shows that they relate to the duration of the disease rather than to cumulative levodopa exposure^12,13^. LID is characterized by involuntary hyperkinetic movements, including chorea, dystonia, and athetosis^14^. Functionally, LID can be explained by the abnormal interference of motor programs within the basal ganglia, disrupting intended motor plans^15^. Several lines of evidence demonstrate an abnormal Hebbian, or spike-timing-dependent, plasticity in the corticostriatal network^16,17^. High-frequency stimulation of cortical afferents induces long-term potentiation (LTP) of corticostriatal synapses in both dyskinetic and non-dyskinetic animals. However, only non-dyskinetic animals exhibit synaptic depotentiation after low-frequency stimulation^18^. In vivo studies in PD patients undergoing deep brain stimulation (DBS) have corroborated this hypothesis, showing an LTP-like effect under levodopa challenge and persistent synaptic potentiation under low-frequency stimulation^19^. Similar findings have been obtained using paired associative stimulation protocols with transcranial magnetic stimulation (TMS) and median nerve stimulation^20^. This evidence suggests that LID involves a form of pathological synaptic plasticity, preventing synaptic downscaling^18,21,22^.

A growing body of evidence in both animal models and human studies supports the hypothesis of altered synaptic plasticity in LID^23,24^. This positions LID as a valuable model for understanding maladaptive neuroplasticity in neurodegeneration. Sleep plays a critical role in regulating synaptic homeostasis by reducing the synaptic burden accumulated during wakefulness^25^. The build-up of synaptic potentiation during the day is counterbalanced by a selective downscaling process that occurs predominantly in early-night sleep, following the homeostatic sleep process. SWA during early SWS serves as the primary electrophysiological marker of this homeostatic function, scaling in response to prior wake duration^26^. The process facilitates neuronal selectivity and enhances signal-to-noise ratios, contributing to memory consolidation and cognitive function^27^. *During sleep*, redundant synaptic connections are eliminated via burst firing characteristic of SWA during intracellular up-down transitions^28,29^. *During wakefulness*, the progressive accumulation of sleep pressure is reflected by increased theta activity^30,31^. Increased theta power has been associated with synaptic potentiation and may represent a compensatory response to synaptic overload. Higher wake theta power correlates with subsequent SWA expression, reinforcing their interconnected role in sleep homeostasis^32,33^.

In our previous work^34^, we demonstrated that dyskinetic PD animals fail to exhibit a physiological reduction in SWA between early and late sleep, suggesting impaired synaptic downscaling. Further, sleep deprivation accelerated the onset of LID in levodopa-treated PD animals, reinforcing the hypothesis that sleep plays a critical role in regulating plasticity mechanisms associated with LID. Our subsequent observational study in human PD patients extended these findings, confirming that while most PD patients experience an overnight reduction in SWA, this pattern is absent in dyskinetic patients^35^. This suggests that impaired SWA-mediated downscaling is uniquely associated with LID, further reinforcing the hypothesis that sleep homeostasis may play a causal role in its pathophysiology. Besides confirming the well-known sleep impairment in PD, our study identified, for the first time, an electrophysiological marker specifically associated with DYS patients. A subsequent study further confirmed a close relationship between SWA-mediated downscaling and the time to the appearance of LID. Specifically, a linear regression model established that the overnight decrease in SWA could predict the time to the emergence of LID^36^.

EEG studies have highlighted a significant increase in low-frequency power, particularly in the theta band, among PD patients. Enhanced 4–6 Hz (low-theta) activity with maximal differences localized over centro-frontal electrodes, suggesting frontal low-frequency dysrhythmia, has been shown^37^. While the most pronounced group-level differences were observed in the 6–9 Hz range, the 4–6 Hz band also showed marked augmentation. Source localization via LORETA identified the generators of this low-theta excess in fronto-insulo-temporal cortical areas, supporting the hypothesis of altered thalamocortical and associative network dynamics in PD^37^. Several EEG studies support the role of theta power augmentation and alpha/theta ratio reduction as markers of PD patients’ cognitive decline. Theta power significantly increases, and the alpha/theta ratio decreases in PD patients with dementia, especially in temporal and occipital regions, with both metrics correlating with disease progression^38^. Similarly, PD patients with mild cognitive impairment (MCI) showed a significant increase in theta power, especially in the left posterior temporal, left occipital, and right frontal regions. A trend toward increased theta is also seen in PD patients without cognitive impairment, suggesting that EEG slowing may appear before clinical symptoms.

Building upon these findings, the present study aims to investigate wake-theta activity in resting states in advanced PD patients with and without dyskinesia. The central focus is to analyze and demonstrate that theta activity in advanced dyskinetic patients is already significantly elevated in the morning and remains stable throughout the day, unlike in controls and other PD groups, where theta activity is lower in the morning and progressively increases as wakefulness extends.

## Results

### Subjects and study design

PD patients were recruited from the outpatient clinics at the Neurocenter of Southern Switzerland and were classified into three groups based on disease progression^39^: (1) early-stage (EPD), comprising patients with a recent diagnosis and without motor fluctuations; (2) advanced without dyskinesia (ADV), comprising advanced patients experiencing end-of-dose or wearing-off phenomenon, motor fluctuation; and (3) advanced dyskinetic (DYS), comprising advanced patients exhibiting motor fluctuations and LID. Healthy age- and sex-matched control subjects were recruited via word of mouth. Inclusion criteria were the following: a diagnosis of PD according to the UK Parkinson’s Disease Society Brain Bank clinical diagnostic criteria; documented or observed LID exceeding 25% of waking hours (where applicable, based on item 4.1 of part IV of the Movement Disorder Society-sponsored revision of the Unified Parkinson Disease Rating Scale (MDS-UPDRS)); stable medication regimen for at least 14 days before study enrollment; age between 30 and 80 years; absence of significant postural instability (Hoehn and Yahr (H&Y) stages II-III); Montreal Cognitive Assessment (MoCA) scores ≥ 26; ability to provide written informed consent^40–43^. Exclusion criteria included comorbid psychiatric disorders, moderate to severe central sleep apnea syndrome, moderate to severe depression, relevant concomitant diseases, or the presence of significant sleep disorders. Demographics of cohorts, clinical assessments, medications, and subjective measures of depression and sleep quality of all patients are reported in Table 1.

**Table 1:** Demographics of cohort, medications, and subjective measures of depression and sleep quality

Groups						
	CTL, *n* = 12	EPD, *n* = 12	ADV, *n* = 13	DYS, *n* = 11	*p*-value	Effect-size
Age, yr	58.83 ± 9.63	64.58 ± 6.84	65.92 ± 9.79	60.91 ± 10.47	0.222	0.094
Gender, % (F/M)	42/58	25/75	8/92	55/45	-	-
Disease duration, yr	NA	3.42 ± 2.19^**a**^	7.54 ± 3.67	8.82 ± 2.96	**3.49e−04**	**0.383**
H&Y	NA	1.25 ± 0.45	1.85 ± 0.38	2.09 ± 0.30	-	-
MDS-UPDRS I	NA	4.325 ± 4.37	6.08 ± 3.82	5.36 ± 3.17	0.700	−0.039
MDS-UPDRS II	NA	5.50 ± 3.78	7.00 ± 3.72	7.00 ± 4.98	0.747	−0.041
MDS-UPDRS III	NA	12.00 ± 8.09	14.46 ± 8.46	11.45 ± 9.06	0.282	0.007
MDS-UPDRS IV	NA	NA	0.69 ± 1.03	2.64 ± 2.80	0.071	0.103
MDS-UPDRS Tot	NA	21.75 ± 14.23	28.23 ± 13.23	26.45 ± 16.05	0.451	−0.020
UDysRS IV	NA	NA	NA	5.80 ± 4.10	NA	NA
UDysRS Tot	NA	NA	NA	26.40 ± 18.28	NA	NA
AIMS	NA	NA	NA	7.64 ± 5.07	NA	NA
LEDD, mg	NA	297.1 ± 169.1^**b**^	863.0 ± 367.8	1078.7 ± 545.4	**7.64e-05**	**0.323**
HADS-A	3.75 ± 2.09	3.83 ± 3.33	3.85 ± 4.12	3.36 ± 2.38	0.907	−0.045
HADS-D	2.17 ± 2.37	2.50 ± 1.57	3.08 ± 3.15	2.64 ± 2.91	0.837	−0.043
ESS	6.25 ± 3.28	5.67 ± 4.08	7.38 ± 4.44	6.27 ± 1.74	0.509	−0.015
PSQI	5.83 ± 2.89	5.00 ± 3.22	7.38 ± 2.10	6.36 ± 4.18	0.298	0.079

*ADV* advanced patients, *AIMS* Abnormal Involuntary Movement Scale, *CTL* control, *DYS* dyskinetic patients, *EPD* early-stage patients, *ESS* Epworth Sleepiness Scale, *HADS-A* Hospital Anxiety and Depression Scale – Anxiety, *HADS-D* Hospital Anxiety and Depression Scale – Depression, *H&Y* Hoehn and Yahr staging, *LEDD* L-dopa equivalent daily dose, *MDS-UPDRS* Movement Disorder Society–sponsored revision of the Unified Parkinson Disease Rating Scale, *NA* not applicable, *PSQI* Pittsburgh Sleep Quality Index, *UDysRS* Unified Dyskinesia Rating Scale.

^a^*p* = 0.0049 versus ADV, and *p* < 0.001 versus DYS, Tukey post hoc test.

^b^*p* < 0.001 versus ADV and DYS, Games-Howell post hoc test.

At the inclusion visit, participants underwent a comprehensive neurological and physical examination. The clinical assessment included MoCA, MDS-UPDRS, H&Y, the Abnormal Involuntary Movement Scale (AIMS), and the Unified Dyskinesia Rating Scale (UDysRS)^44,45^. Sleep-related symptoms and mood disturbances were assessed using validated questionnaires, including the Hospital Anxiety and Depression Scale (HADS) for mood disorders and the Epworth Sleepiness Scale (ESS), Pittsburgh Sleep Quality Index (PSQI), and Scales for Outcomes in Parkinson’s Disease–Sleep (SCOPA-Sleep) for sleep disturbances^46–48^. To evaluate sleep-wake patterns, participants were equipped with inertial sensors (GENEActiv) for one-week. They were instructed to maintain a regular sleep-wake schedule, and compliance was monitored via inertial sensor recordings. Following this one-week monitoring period, participants completed two short wakefulness recordings (each lasting 30 minutes), during which they also performed a go/no-go task. The first recording took place in the morning, and the second occurred nine hours later in the evening. All recordings were conducted at the participant’s home and included high-density electroencephalography (hd-EEG) during wakefulness (eyes open, eyes closed, and task performance) in the morning and evening. All PD patients were administered antiparkinsonian drugs at a stable and optimized dosage, as determined by the enrolling neurologist (S.G., I.B.). All the patients were in an ON state during hd-EEG recordings, defined as the clinically effective phase following medication intake, and none exhibited motor fluctuations or wearing-off phenomena at the time of assessment.

**Fig. 1:**
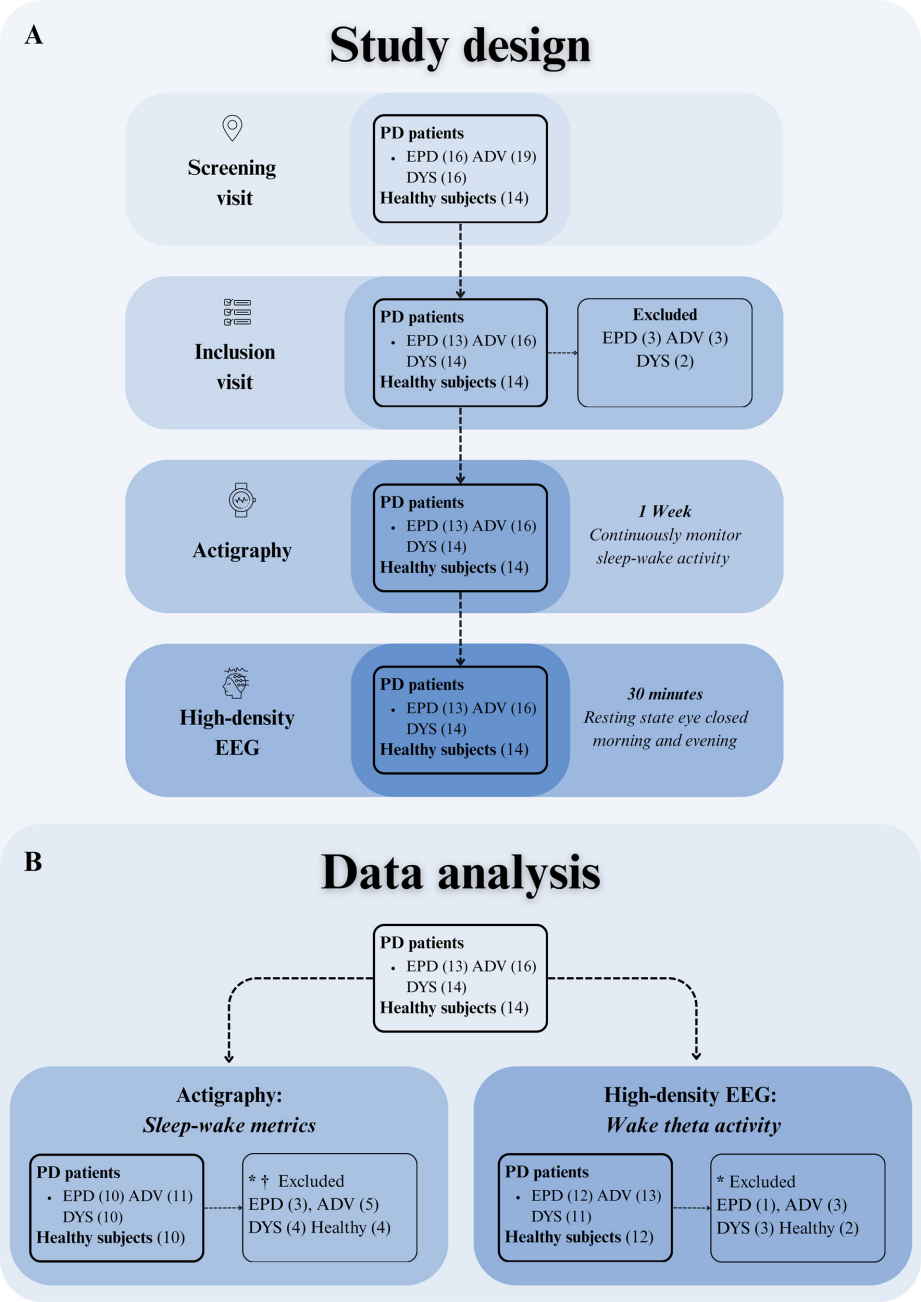
Study design and data analysis. **A** Schematic flow chart showing participant enrollment, assessments, and data analysis. Following screening, eight patients (3 EPD, 3 ADV, 2 DYS) were excluded per the inclusion/exclusion criteria. At inclusion, participants underwent neurological/physical examinations and were equipped with inertial sensors for actigraphy monitoring. After one week of actigraphy, two 30-minute wakefulness sessions, eyes-closed resting states, i.e., morning and evening, were conducted using high-density EEG (hd-EEG), spaced approximately nine hours apart. **B** * Exclusions were applied separately for actigraphy and hd-EEG analyses. Participants with non-compliant sleep-wake cycles (via inertial sensors) were excluded from actigraphy-derived metrics. For hd-EEG, exclusions were based on poor data quality or technical artifacts in morning/evening resting-state recordings. † Subjects excluded from actigraphy analysis were additionally screened for hd-EEG completeness to ensure the cohort retained only individuals with high-quality data across both modalities for integrated sleep-wake and EEG assessments. ADV advanced patients without dyskinesia, CTL controls, DYS advanced patients with dyskinesia, EPD early-stage patients.

In Fig. 1 we report an overview of the study design, with a schematic flow chart showing participant enrollment, assessments, and data analysis. A total of 65 individuals were initially enrolled (14 healthy controls, 16 EPD, 19 ADV without dyskinesia, and 16 DYS). Following screening procedures, eight patients (3 EPD, 3 ADV, and 2 DYS) were excluded based on predefined inclusion and exclusion criteria, resulting in 14 controls, 13 EPD, 16 ADV, and 14 DYS participants proceeding to data acquisition. For high-density EEG analyses, additional exclusions were made due to poor data quality or technical artifacts in the morning or evening resting-state recordings. The final hd-EEG dataset therefore, included 12 controls, 12 EPD, 13 ADV, and 11 DYS participants. Participants showing non-compliant sleep-wake cycles, as detected by inertial sensors, were excluded from actigraphy-derived sleep metrics. Those excluded from actigraphy analysis were also reassessed to ensure completeness of hd-EEG data, thereby retaining only participants with high-quality data across both modalities for integrated sleep–wake and EEG analyses. Consequently, the subset used for joint slep metric–EEG correlation analyses included 10 controls, 10 EPD, 11 ADV, and 10 DYS participants.

### Demographic and clinical variables

A detailed examination of demographic and clinical characteristics, as presented in Table 1, revealed that the study groups were generally well-matched across several key variables, including age, scores on the MDS-UPDRS scales, as well as subjective assessments of depression and sleep quality. Statistical analysis confirmed the absence of significant differences between groups for these parameters, with all comparisons yielding *p* > 0.05. Disease duration varied significantly among the groups (*p* = 3.49 × 10⁻⁴). Post hoc analyses clarified that EPD patients had a markedly shorter disease duration compared to both the ADV (*p* = 0.0049) and DYS (*p* < 0.001) groups. This pattern underscores the clinical distinction between newly diagnosed patients and those with more advanced disease stages, particularly those experiencing motor complications. Levodopa equivalent daily dose (LEDD) also clearly emerged as a differentiating factor (*p* = 7.64 × 10⁻⁵). EPD patients exhibited substantially lower LEDD compared to both ADV and DYS groups (*p* < 0.001 for both comparisons), reflecting the expected escalation in dopaminergic therapy as the disease progresses and motor fluctuations become more prominent. Importantly, when comparing the two advanced PD subgroups – ADV and DYS – no significant differences were observed in either disease duration or LEDD, suggesting that these groups were closely matched in terms of disease progression and medication exposure. The overlap in clinical variables and medications between ADV and DYS groups provides a robust foundation for subsequent analyses focused on distinguishing features, such as sleep and electrophysiological measures, which may underline the development of dyskinesia in PD.

### Actigraphy: Sleep-wake metrics

Analysis of weekly actigraphy data, as summarized in Table 2, reveals clear group differences in objective sleep-wake parameters. DYS experienced the most substantial disruptions in sleep architecture. Notably, their sleep efficiency was markedly reduced when compared to both healthy controls (*p* < 0.001) and EPD patients (*p* < 0.05), while the ADV group showed intermediate levels of impairment, with sleep efficiency also significantly lower than controls (*p* < 0.05). This pattern suggests a gradation of sleep quality deterioration corresponding to disease progression and the emergence of motor complications. Sleep latency, representing the time required to fall asleep, was significantly prolonged in both DYS and ADV groups relative to controls (*p* < 0.05 for both), indicating increased difficulty in initiating sleep among patients with more advanced PD. Furthermore, the sleep regularity index, a measure of the consistency of sleep-wake timing, was lowest in the DYS group and significantly differed from controls (*p* < 0.05), highlighting a pronounced irregularity in their daily sleep patterns. Another relevant finding was observed in the wake-after-sleep onset metric, which quantifies nocturnal awakenings. DYS patients exhibited nearly double the WASO compared to controls (*p* = 0.01), reflecting fragmented sleep and frequent nighttime disruptions. In terms of total sleep time, both ADV and DYS groups slept less overall, with the reduction reaching statistical significance for ADV compared to controls (*p* < 0.05). Interestingly, despite shorter sleep duration, DYS patients spent more time in bed than those in the ADV group (*p* < 0.05), possibly reflecting attempts to compensate for poor sleep quality. Taken together, these actigraphy findings illustrate a trajectory of sleep disturbance in PD, with DYS patients displaying the most severe impairments across multiple domains of sleep continuity, regularity, and efficiency.

**Table 2:** Descriptive actigraphy data weekly

Groups						
	CTL, *n* = 10	EPD, *n* = 10	ADV, *n* = 11	DYS, *n* = 10	*p*-value	Effect-size
eIS, %	0.66 ± 0.09	0.64 ± 0.13	0.60 ± 0.13	0.64 ± 0.08	0.644	0.043
eIV, %	0.68 ± 0.22	0.71 ± 0.15	0.91 ± 0.18	0.71 ± 0.23	**0.037**	**0.202**
eSE, %	0.86 ± 0.04^a^	0.80 ± 0.06^b^	0.75 ± 0.08	0.68 ± 0.09	**1.49e−04**	**0.467**
eSL, h	0.21 ± 0.06^**c**^	0.40 ± 0.20	0.48 ± 0.22	0.61 ± 0.36	**0.002**	**0.307**
eSRI, %	60.59 ± 13.72^d^	53.27 ± 10.68	50.97 ± 13.84	43.17 ± 7.63	**0.020**	**0.231**
eTIB, h	7.85 ± 0.61	8.16 ± 0.90	7.31 ± 1.15^**e**^	8.75 ± 1.35	**0.025**	**0.221**
eTST, h	6.74 ± 0.48^f^	6.48 ± 0.71	5.51 ± 1.16	5.93 ± 1.27	**0.028**	**0.215**
eWASO, h	0.90 ± 0.32^g^	1.27 ± 0.61	1.31 ± 0.57	2.21 ± 0.70	**0.002**	**0.307**

*ADV* advanced patients, *AIMS* Abnormal Involuntary Movement Scale. *CTL* control, *DYS* dyskinetic patients, *EPD* early-stage patients, *eIS* Interdaily Stability, *eIV* Intradaily Variability, *eSE* Sleep Efficiency, *eSL* Sleep Latency, *eSRI* Sleep Regularity Index, *eTIB* Time In Bed, *eTST* Total Sleep Time, *eWASO* Wake After Sleep Onset.

^a^*p* < 0.05 versus ADV and p < 0.001 versus DYS, Dunn post hoc test.

^b^*p* < 0.05 versus DYS, Dunn post hoc test.

^c^*p* < 0.05 versus ADV and DYS, Dunn post hoc test.

^d^*p* < 0.05 versus DYS, Tukey post hoc test.

^e^*p* < 0.05 versus DYS, Tukey post hoc test.

^f^*p* < 0.05 versus ADV, Tukey post hoc test.

^g^*p* = 0.01 versus DYS, Dunn post hoc test.

### High-density EEG: Wake theta activity

**Fig. 2:**
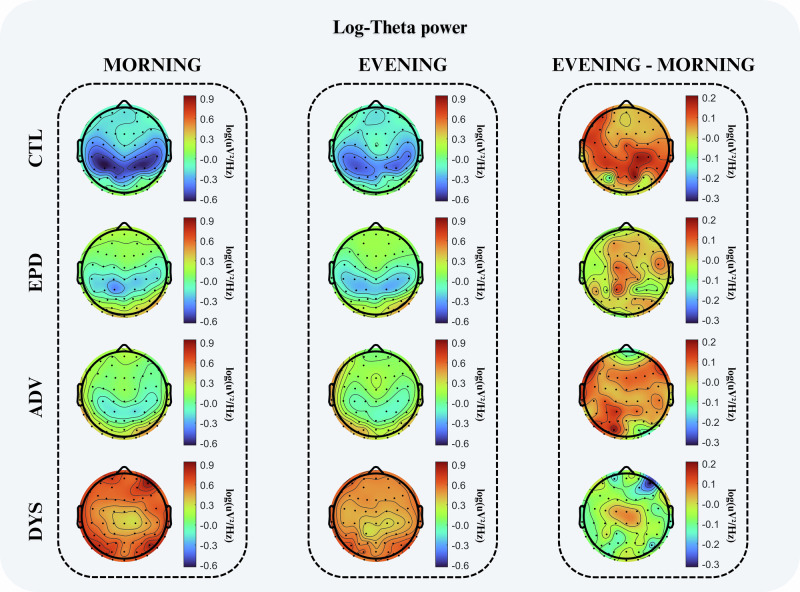
Morning, evening, and diurnal dynamics of theta power across groups. Topographical maps illustrate log-transformed theta power (4–8 Hz, units: log(uV^2^/Hz)) during morning, evening, and their diurnal difference (evening minus morning) across control (CTL), early-stage (EPD), advanced (ADV), and dyskinetic (DYS) groups. Morning and evening scales were normalized independently to the global minimum and maximum values across all groups. CTL showed the lowest morning theta power, with higher values in EPD and ADV, and the highest levels in DYS. In the diurnal maps, CTL, EPD, and ADV displayed a visible increase in theta power from morning to evening, whereas DYS showed only minimal diurnal change, qualitatively indicating reduced daytime build-up of theta activity. ADV advanced patients without dyskinesia, CTL controls, DYS advanced patients with dyskinesia, EPD early-stage patients.

A qualitative analysis of high-density EEG recordings, illustrated in Fig. 2, reveals emerging patterns of log-transformed theta power (4–8 Hz) across the study groups during both morning and evening resting states, as well as in the diurnal difference. Healthy controls showed the lowest morning theta power, with higher values in the EPD and ADV groups and the highest levels in the DYS group. When examining the diurnal accumulation of theta (evening minus morning), CTL, EPD, and ADV groups all showed a visible increase in theta power from morning to evening, compatible with a physiological daytime build-up of theta activity. In contrast, the DYS group showed only minimal diurnal change, suggesting a disruption of this expected increase in wake-related theta activity.

**Fig. 3:**
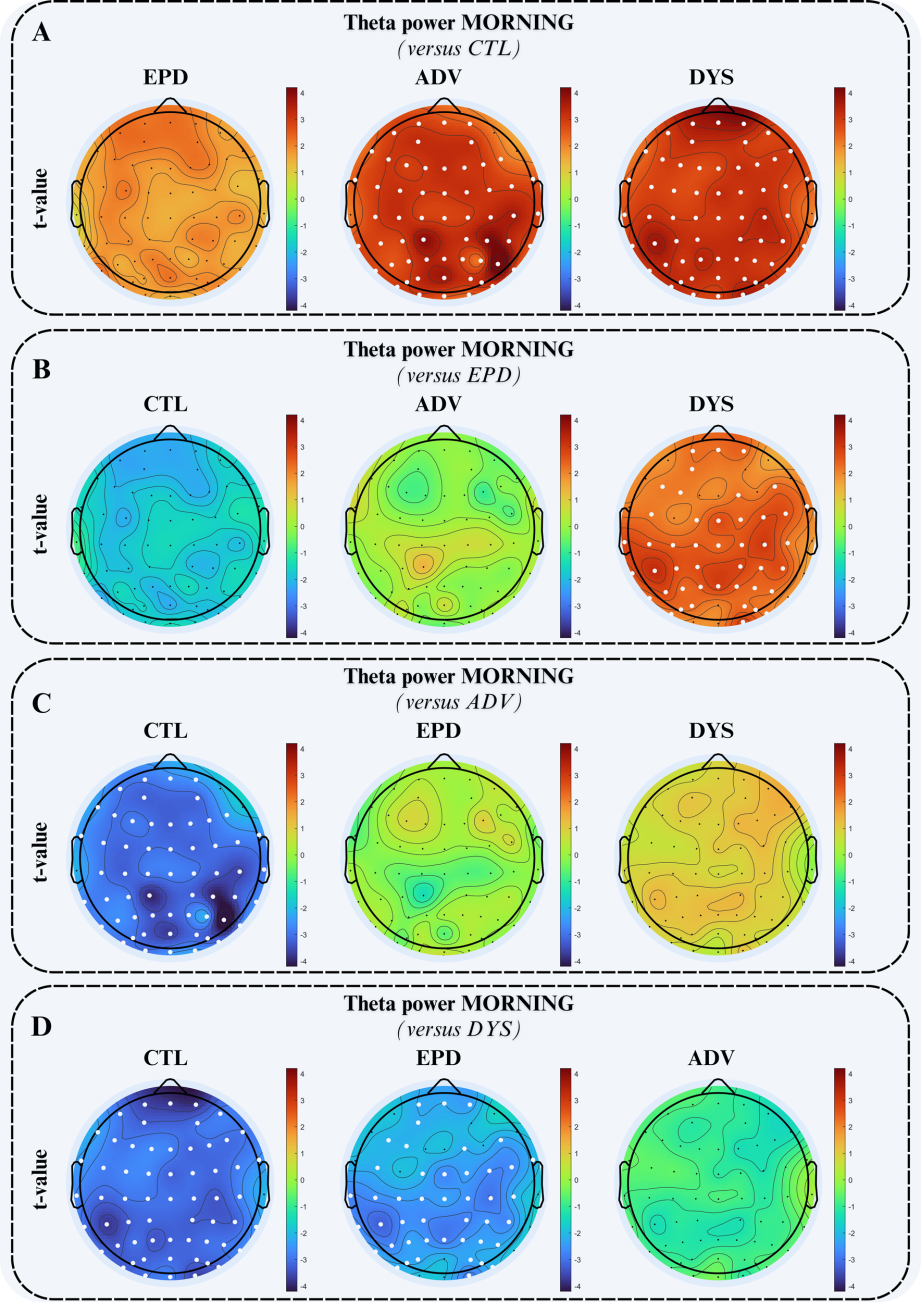
Morning theta power differences across groups. Group-level topographical maps of t-values from MLR models of log-transformed morning theta power (4–8 Hz, units: log(uV^2^/Hz)), adjusted for age and gender. Maps show the direction and magnitude of group differences when contrasting each group with **A** CTL, **B** EPD, **C** ADV, and **D** DYS as the baseline (warm colors: higher theta than baseline; cool colors: lower theta than baseline). White dots mark electrodes with TFCE-corrected significant effects (*p* < 0.05). ADV advanced patients without dyskinesia, CTL controls, DYS advanced patients with dyskinesia, EPD early-stage patients.

The quantitative analysis of morning theta power changes is shown in Fig. 3. Region-wise comparisons for morning theta power, including all contrasts among CTL, EPD, ADV, and DYS, are also summarized in Supplementary Table S1. MLR models with CTL as baseline (Fig. 3A), adjusted for age and gender, showed significantly higher morning theta power in both ADV and DYS groups at the whole-scalp level (ADV versus CTL: *p* = 0.008, Cohen’s d = 1.10; DYS versus CTL: *p* = 0.006, Cohen’s d = 1.54), with increases involving prefrontal, temporal, sensorimotor, parietal, occipital, and temporo-parietal regions for both groups. In contrast, EPD patients did not differ significantly from CTL after TFCE correction, although effect sizes were consistently positive and in the moderate range, suggesting an intermediate increase in morning theta. To further delineate morning theta differences among patient subgroups, additional TFCE-corrected MLR models were estimated using EPD, ADV, and DYS as alternative reference groups (Fig. 3B–D). These parameterizations exploit EPD and ADV as disease‑stage baselines, enabling formal pairwise tests among PD subgroups in addition to the contrasts with CTL. When ADV was compared with EPD, no significant regional differences emerged, and effect sizes remained small (Cohen’s d generally below 0.35), indicating largely overlapping morning theta levels between these two stages. Contrasts between DYS and EPD showed significant spatially extended increases in morning theta in DYS (whole scalp and multiple regions, including motor, parietal, sensorimotor, and temporo‑parietal areas; p values from 0.03 to 0.05 with Cohen’s d from 0.7 to 1.0). On the other hand, comparisons between DYS and ADV did not reach significance but still yielded small‑to‑moderate effect sizes. Overall, these morning MLR results indicate a broad theta elevation in both advanced groups relative to controls, with DYS patients showing an additional gain over EPD. In Supplementary Fig. S1, we also report the same theta morning analysis from unadjusted models (no age/sex covariates). EPD versus CTL differences become here more pronounced and approach significance in several regions. The overall pattern of elevated morning theta in advanced PD relative to controls remains.

**Fig. 4:**
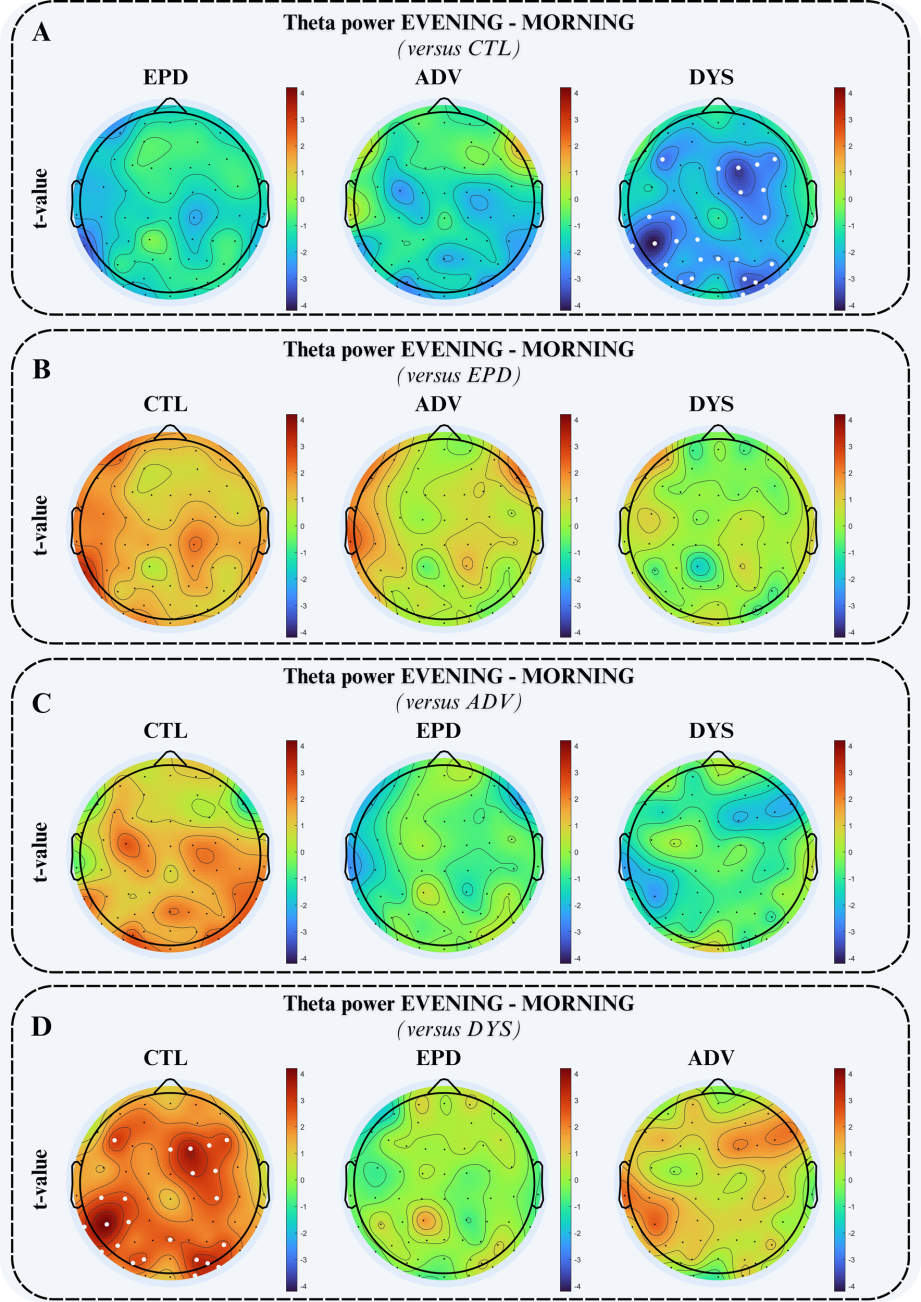
Diurnal theta power differences across groups. Group-level topographical maps of t-values from LMMs of diurnal theta change (evening minus morning, 4–8 Hz, units: log(uV^2^/Hz)), adjusted for age and gender. Maps show the direction and magnitude of group differences when contrasting each group with **A** CTL, **B** EPD, **C** ADV, and **D** DYS as the baseline (warm colors: greater diurnal increase than baseline; cool colors: reduced diurnal increase than baseline). White dots indicate electrodes with TFCE-corrected significant effects (*p* < 0.05). ADV advanced patients without dyskinesia, CTL controls, DYS advanced patients with dyskinesia, EPD early-stage patients.

The quantitative analysis of diurnal theta power changes, defined as the difference between evening and morning sessions, is shown in Fig. 4. Region-wise comparisons for diurnal theta power, including all contrasts among CTL, EPD, ADV, and DYS, are also summarized in Supplementary Table S2. LMMs models with CTL as baseline (Fig. 4A), adjusted for age and gender, showed no significant diurnal effects for EPD or ADV relative to CTL after TFCE correction (all *p* ≥ 0.07 and whole‑scalp Cohen’s d from −0.82 to −0.91). In contrast, DYS patients showed a markedly reduced theta increase over the day compared with CTL (whole scalp, *p* = 0.009, Cohen’s d = −1.57), with significant negative effects in prefrontal, sensorimotor, motor, parietal, occipital, and temporo‑parietal/ occipito‑temporal regions (*p* values from 0.004 to 0.03 and Cohen’s d from −1.1 to −1.6). To further explore whether diurnal impairments differentiated the PD subgroups, the same age‑ and sex‑adjusted LMMs were re‑estimated using EPD, ADV, and DYS as alternative reference groups (Fig. 4B–D), thereby providing all pairwise contrasts among PD subgroups in addition to the CTL‑based models. When EPD was set as baseline, neither ADV nor DYS showed TFCE‑significant diurnal differences (all *p* ≥ 0.17), despite small‑to‑moderate effect sizes for some regions (e.g., ADV versus EPD temporal cortex, Cohen’s d = 0.78). Similarly, comparisons with DYS as baseline did not reveal significant regional contrasts with ADV or EPD (all *p* ≥ 0.11), and effect sizes remained in the small‑to‑moderate range. Overall, these diurnal results indicate that a blunted daytime build‑up of theta power is detectable only when contrasting DYS with CTL at the group level, with ADV and EPD showing no TFCE‑significant diurnal differences relative to CTL, whereas variability within the PD subgroups and potential daytime confounds likely obscure possible phenotype‑specific differences in PD diurnal dynamics. Supplementary Fig. S2 reports the corresponding unadjusted LMM results (no age/sex covariates), which closely resemble the age/sex‑adjusted maps and show that the markedly reduced diurnal theta build‑up in DYS versus CTL is essentially unchanged, suggesting that this core effect is not driven by age/sex differences between groups.

### Correlation analysis

**Fig. 5:**
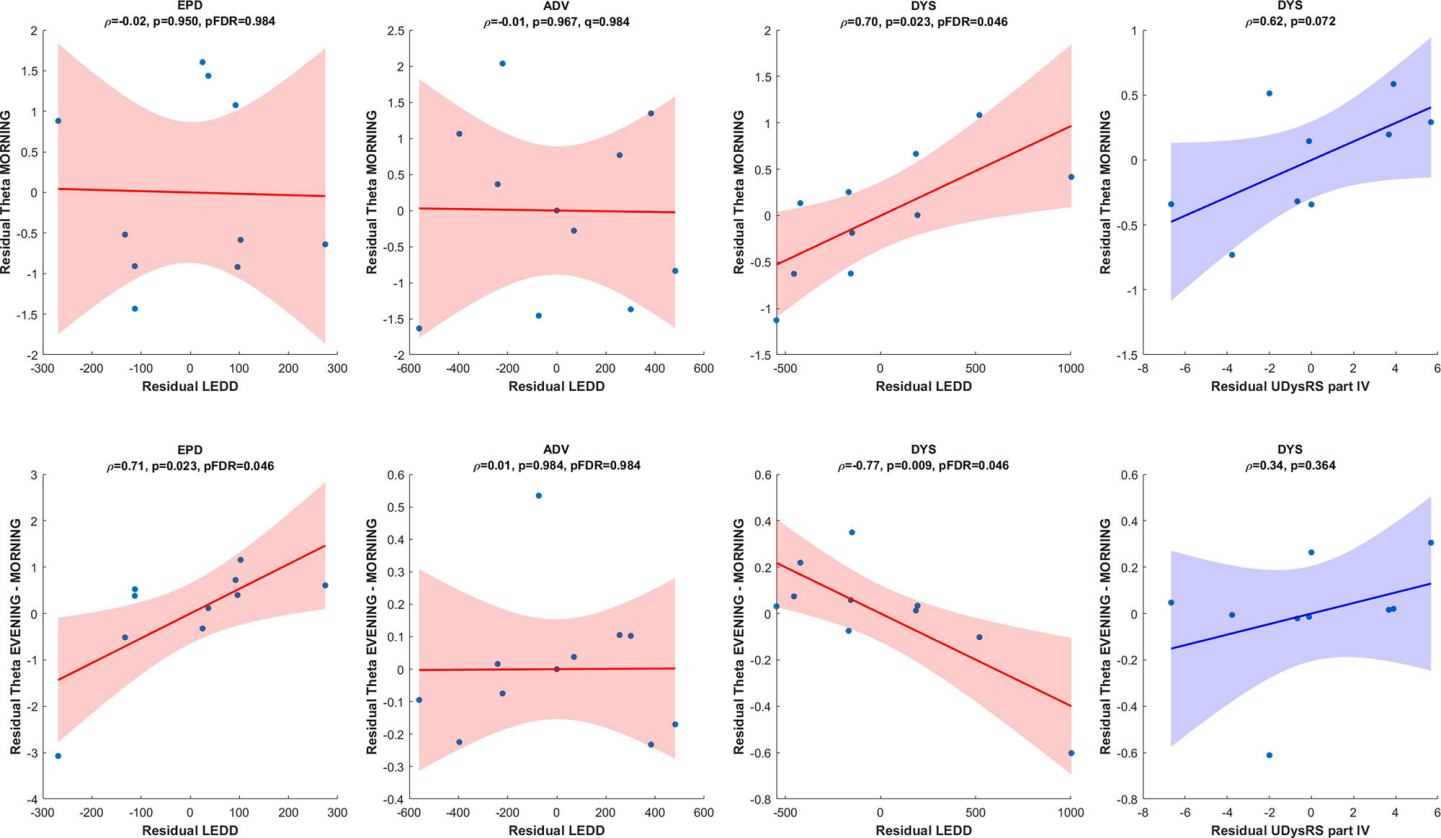
Correlation between theta power, dopaminergic medication load, and dyskinesia severity. Scatterplots in red illustrate the correlations between residual log-transformed theta power measures (top row: morning theta; bottom row: diurnal theta change, defined as evening minus morning) and residual levodopa equivalent daily dose (LEDD), all adjusted for age and gender, in early-stage (EPD), advanced (ADV), and dyskinetic (DYS) patients. Scatterplots in blue illustrate the correlations between residual log-transformed theta power measures (top row: morning theta; bottom row: diurnal theta change) and residual Unified Dyskinesia Rating Scale (UDysRS) Part IV scores, adjusted for age and gender, in DYS patients. For LEDD-related analyses, six subgroup correlations (three groups × two theta outcomes: morning and evening minus morning) were treated as a single family of tests, and Benjamini–Hochberg false discovery rate (BH–FDR) correction was applied; both uncorrected *p* values and FDR-adjusted *p* values are reported in the panel labels. In DYS patients, higher LEDD was associated with higher morning theta power and a smaller diurnal theta increase, whereas no significant LEDD–morning theta correlations were observed in EPD or ADV, and a positive LEDD–diurnal theta association emerged only in EPD. In DYS patients, morning theta showed a positive but statistically inconclusive trend with UDysRS Part IV, and no clear association was present for the diurnal theta change. Each plot displays the best-fit regression line and shaded area representing the 95% confidence interval. ADV advanced patients without dyskinesia, CTL controls, DYS advanced patients with dyskinesia, EPD early-stage patients, LEDD levodopa equivalent daily dose, UDysRS Part IV Unified Dyskinesia Rating Scale Part IV. The number of DYS patients in this correlation analysis is *n* = 9 (UDysRS value was not available for one subject).

Correlation analysis based on age- and sex-adjusted residuals is summarized in Fig. 5. In the DYS subgroup, higher LEDD was associated with greater morning theta power (*ρ* = 0.70, *p* = 0.023, pFDR = 0.046, 95% CI [0.13, 0.92]) and with a smaller diurnal theta increase (evening minus morning; *ρ* = −0.77, *p* = 0.009, pFDR = 0.046, 95% CI [−0.94, −0.28]). In contrast, no significant correlations between LEDD and morning theta were observed in the EPD or ADV groups (pFDR = 0.98). A positive association between LEDD and diurnal theta change emerged in EPD (*ρ* = 0.71, *p* = 0.023, pFDR = 0.046, 95% CI [0.14, 0.92]), but this effect was not present in the other groups. Within the DYS subgroup, morning theta also showed a positive but non‑significant trend with dyskinesia severity indexed by UDysRS Part IV (*ρ* = 0.62, *p* = 0.072, 95% CI [−0.07, 0.91]), while no clear association was found for the diurnal theta change (*ρ* = 0.34, *p* = 0.36, 95% CI [−0.41, 0.82]). Taken together, these small‑sample correlations are consistent with the possibility that, in dyskinetic patients, higher dopaminergic medication load may be linked to higher morning theta and a reduced capacity for further theta build‑up across the day, with dyskinesia severity showing a parallel but statistically inconclusive relationship. These patterns remain exploratory and require confirmation in larger cohorts.

Partial correlations between theta measures and actigraphy-derived sleep metrics are reported in Supplementary Figures S3 and S4. In the EPD subgroup, trends suggested that lower sleep efficiency and greater wake after sleep onset were associated with higher morning theta (SE: *ρ* = −0.63, *p* = 0.052, pFDR = 0.312, 95% CI [−0.90, 0.00]; WASO: *ρ* = 0.75, *p* = 0.013, pFDR = 0.075, 95% CI [0.23, 0.94]), and with smaller diurnal theta increases at higher WASO (*ρ* = −0.57, *p* = 0.087, pFDR = 0.174, 95% CI [−0.88, 0.10]). In the DYS subgroup, higher WASO tended to relate to smaller diurnal theta increases (*ρ* = −0.63, *p* = 0.050, pFDR = 0.149, 95% CI [−0.90, −0.01]), while morning theta correlations were weak. Overall, no FDR-corrected significant associations emerged across theta–sleep metric combinations, and additional correlations between actigraphy metrics and clinical scores (e.g., UDysRS) were uniformly non‑significant.

These correlation findings, obtained from small groups and accompanied by wide confidence intervals, are here presented as hypothesis‑generating only. Actigraphy-based measures primarily capture macrostructural aspects of sleep-wake continuity rather than underlying neurophysiological mechanisms, and more in-depth measures of sleep-wake coupling will require longitudinal studies combining full‑night polysomnography with repeated waking EEG recordings, as planned in our additional ongoing work.

## Discussion

The study provides the first insight that LID in PD is characterized by pronounced sleep-wake macrostructural disruption and markedly altered wake theta dynamics. Our contributions can be summarized as follows: (i) We identified pronounced sleep dysregulation in dyskinetic patients, as reflected by actigraphy-derived sleep macrostructure metrics showing reduced sleep efficiency, prolonged sleep latency, and increased nocturnal awakenings compared to other PD subgroups and controls. (ii) We found that only dyskinetic patients, compared to controls, showed both a significant increase in theta power in the morning and an absence of diurnal scaling. These effects were particularly evident over prefrontal, temporal, and occipital regions, suggesting a saturated, and consequently, stable theta profile unique to the dyskinetic group. (iii) We found associations between theta abnormalities and clinical features of dyskinesia, showing that theta saturation correlates with dopaminergic medication load, and a positive trend with dyskinesia severity, highlighting its potential role as a biomarker of maladaptive plasticity.

Examining the nocturnal activity, we confirm the presence of an impaired sleep structure in LID, as indicated by actigraphy-derived metrics that indirectly reflect the macrostructural consequences of disrupted sleep regulation. While such measures, including estimated sleep efficiency and estimated total sleep time, have been previously used to compare PD patients to healthy controls^49^, this study is the first to clearly show a progressive sleep fragmentation across PD subgroups using actigraphy-derived measures. To the best of our knowledge, the only comparable study is our previous work, which was conducted on a smaller cohort and used different hardware and software configurations, leading to non-significant differences between groups^35^. In contrast, the current analysis reveals a novel stratification of sleep-wake dysregulation across PD stages, with dyskinetic DYS patients exhibiting the most severe impairments. Sleep efficiency is significantly reduced in DYS compared to CTL and EPD, while ADV patients show intermediate deficits. The disrupted sleep regularity in DYS and elevated wake-after-sleep onset (nearly doubled compared to CTL) suggest compounded macrostructural sleep deficits. These findings align with previous reports of fragmented sleep initiation in PD, and with our earlier work indirectly linking sleep fragmentation to the severity of LID^35,49^. Our results further extend the literature by revealing a dissociation between subjective sleep assessments, which showed no significant group differences in PSQI or ESS scores, and objectively measured sleep impairments. DYS patients spent more time in bed, yet achieved less total sleep time compared to controls, suggesting a mismatch between sleep opportunity and actual restorative sleep. The dissociation between actigraphy and subjective measures (i.e., PSQI or ESS) is consistent with prior validation studies showing limited concordance between perceived sleep quality and objective indices in clinical populations and underscores the complementary value of actigraphy and questionnaires^50,51^.

To test our hypothesis that dyskinetic patients exhibit maladaptive plasticity not only during sleep, but also across wakefulness, we next examined theta activity, i.e., an electrophysiological marker of synaptic potentiation during the daytime. Theta activity is known to increase progressively with awake time, and it has been proposed as a surrogate marker of accumulating sleep pressure and synaptic load^26^. We show that DYS patients exhibit a unique electrophysiological signature: saturated morning theta power, which remains stable throughout the day, contrasting with the physiological theta accumulation observed in EPD and ADV patients. This theta profile, maximal at wake onset and spatially concentrated in prefrontal, temporal, and occipital regions, may indicate a disruption in the brain’s ability to modulate synaptic plasticity throughout the sleep-wake cycle^52^. The observed pattern in DYS patients may reveal a pathological deviation from the synaptic homeostasis mechanisms^26^. DYS patients exhibit elevated morning theta power, contrasting with the expected post-sleep synaptic reset. This result parallels observations made in rodent models, where chronic L-dopa abolishes SWA-dependent depotentiation, locking corticostriatal synapses in a hyper-potentiated state^18,34^. The frontal predominance of theta abnormalities mirrors LID’s corticostriatal pathophysiology, with frontal theta morning presenting a positive trend with UDysRS Part IV scores^20^.

Our findings converge with existing literature on the role of fronto-striatal circuitry in LID. The frontal cortex, especially prefrontal and supplementary motor areas, plays a critical role in modulating dopaminergic tone and motor control. Prior neuroimaging studies have shown that hyperactivity and structural alterations in these regions correlate with dyskinesia severity^53,54^. The observed occipito-temporal involvement, though less expected, aligns with recent evidence that posterior cortical areas contribute to sensorimotor integration and visual-motor feedback loops, which are often disrupted in advanced PD and LID. Notably, these regions are also known to participate in global arousal regulation and cognitive control, further linking our findings to broader dysfunctions in homeostatic sleep-wake plasticity.

The relationship between electrophysiological markers, i.e., theta activity, and clinical variables reveals additional insights. Partial correlation analyses demonstrated that a significant positive correlation between morning theta power and LEDD was present only in DYS patients, linking higher dopaminergic medication load to greater frontal baseline theta in this subgroup. In addition, within DYS, higher LEDD was also associated with a smaller diurnal theta increase, indicating that chronic levodopa exposure is related not only to elevated morning theta but also to a reduced capacity for further daytime theta build-up. In contrast to the LEDD effects, no significant correlations were found between morning theta power and disease duration in either the ADV or DYS groups, and LEDD-theta associations were absent in ADV and followed an opposite pattern for diurnal change in EPD, where higher LEDD was related to larger theta increases across the day. Additionally, there were no significant differences between the ADV and DYS groups regarding disease duration or LEDD. These findings suggest that the unique theta alterations observed in DYS patients cannot be attributed only to disease progression or higher medication dosage. Instead, the impaired theta profile in DYS may be a specific pathophysiological sleep-related feature of dyskinesia, possibly reflecting a dysfunctional interplay between chronic dopaminergic stimulation and impaired sleep-wake synaptic regulation. These results suggest that levodopa influences theta activity in DYS, and, while speculative, higher theta activity in the morning may reflect a failure in synaptic plasticity, i.e., synaptic downscaling and impaired regulation of daytime theta dynamics. Partial correlations between theta measures and actigraphy-derived sleep metrics provided suggestive evidence for the hypothesis above. In EPD, trends indicated that lower estimated SE and higher WASO were associated with higher morning theta and smaller diurnal theta increases, suggesting that sleep fragmentation may be linked to altered wake theta in early disease. In DYS, higher WASO tended to relate to smaller diurnal theta increases, indicating that more fragmented sleep may be related to a reduced capacity for daytime theta build-up in this subgroup. These patterns suggest that both chronic dopaminergic exposure and sleep-wake disruption may contribute to altered theta dynamics in dyskinesia.

Several limitations of this study should be acknowledged. First, actigraphy provides reliable macrostructural estimates of sleep continuity but cannot capture sleep microstructure, such as SWA or sleep spindles, nor can it disentangle specific NREM stages, limiting the ability to map theta changes into specific SWS-related processes. Second, we lacked consistent data regarding napping behavior between the morning and evening hdEEG recordings. Neither objective (e.g., actigraphy) nor subjective (e.g., sleep diaries) nap information was systematically collected across all patients. This omission could confound the interpretation of diurnal theta dynamics, as daytime sleep episodes may influence cortical excitability and synaptic load. Third, the sample sizes for subgroup partial correlation analyses were small, as reflected by wide confidence intervals even for relatively large correlation coefficients, and all such correlations are presented as exploratory and must be interpreted cautiously. Finally, the absence of strong, consistent correlations between theta and sleep metrics underlines the limitations of actigraphy as a proxy for sleep microstructure and indicates that macrostructural measures alone may not capture the full neurophysiological substrates of sleep-dependent regulation. Rather than demonstrating a definitive bidirectional causal failure, our results indicate that dyskinetic patients show a distinctive combination of fragmented sleep and elevated, poorly upscaling daytime theta activity, pointing to a disruption of coordinated sleep-wake plasticity that warrants further mechanistic investigation.

Despite these limitations, the convergence of actigraphy, EEG, and clinical correlation findings points to impaired wake theta dynamics as a promising marker of dyskinesia-related network dysfunction within a broader sleep-wake plasticity framework. Ongoing longitudinal work in the same cohort, which includes full-night polysomnography across groups and repeated waking EEG recordings, will be essential to test within-subject night-to-day coupling of SWA and theta activity, to comprehensively characterize the temporal dynamics of sleep-wake synaptic regulation. This future study will help clarify whether wake theta alterations primarily reflect a marker of dyskinesia and dopaminergic load, an index of more global homeostatic imbalance, or a combination of both, and whether they can be modulated by interventions targeting sleep and slow-wave activity. In this perspective, sleep-focused strategies aimed at improving slow-wave sleep and stabilizing sleep-wake continuity remain an attractive, though yet unproven, avenue to restore synaptic equilibrium in PD and potentially mitigate the development or expression of levodopa-induced dyskinesia.

## Methods

### Actigraphy: Sleep-wake metrics

Wrist-worn actigraphy is a well-established method for objectively assessing sleep-wake patterns. In this study, GENEActiv inertial sensors were used to continuously monitor activity from the participant’s nondominant wrist over a one-week period. Data were recorded at a sampling frequency of 50 Hz and subsequently preprocessed using the open-source R package *GGIR* (version 3.1.5). GGIR is a research community-driven software developed for the extraction of physical activity and sleep-related metrics from raw multi-day accelerometer recordings^55^. To obtain a picture of sleep regulation and activity patterns, several key metrics were derived as weekly averages. Participants were instructed to complete a sleep log during the monitoring period, providing information such as ‘light off’ and ‘got up’ times, with clear written instructions on how to accurately report each entry. The following sleep and activity-related metrics were extracted (i.e., *estimated -e*) from the GGIR output reports, based on validated algorithms implemented within the package^56,57^:

- Inter-daily stability (eIS), i.e., a metric assessing the degree to which an individual maintains a stable rhythm of daily activity across multiple days, with values ranging from 0 to 1, where higher values indicate greater stability.
- Intra-daily variability (eIV), i.e., an index quantifying the fragmentation and fluctuation of activity levels within a single day, highlighting the extent to which activity patterns shift over short periods.
- Sleep efficiency (eSE), i.e., the ratio of total sleep time to time in bed, providing an indication of how effectively time in bed was used for sleep.
- Sleep latency (eSL), i.e., the duration between ‘light off’ and ‘fell asleep,’ serving as an indicator of sleep initiation difficulties.
- Sleep regularity index (eSRI), i.e., a measure quantifying the stability of sleep-wake patterns over consecutive 24-hour periods, providing insight into the consistency of the individual’s sleep schedule.
- Time in bed (eTIB), i.e., the total duration between ‘light off’ and ‘got up’ times, reflecting the period intended for sleep.
- Total sleep time (eTST), i.e., the total duration spent asleep, derived from epoch-by-epoch sleep/wake classification, excluding the periods of sleep latency and wake episodes occurring between ‘fell asleep’ and ‘wake up” times.
- Wake after sleep onset (eWASO), i.e., the total duration of wakefulness occurring between ‘fell asleep’ and ‘wake up’, reflecting sleep fragmentation.

The actigraphy-derived measures allow for an objective comparison of sleep patterns across different groups. Given our prior research, we aim to explore whether these sleep-related metrics provide further evidence of distinct sleep-related alterations associated with DYS.

### High-density EEG: Wake theta activity

High-density EEG recordings were acquired using ANT Neuro amplifiers and 64-channel electrode caps placed according to the 10–20 international system, with the impedance of each active electrode kept below 20 kΩ. Data were collected during two separate resting-state conditions, eyes closed, i.e., morning and evening. Raw hd-EEG signals were first band-stop filtered at 50 Hz using a Hamming-windowed sinc finite impulse response filter to remove power line noise. A high-pass filter with a cutoff of 0.5 Hz and a low-pass filter at 100 Hz, both implemented with Hamming-windowed FIR filters, were then applied to attenuate slow drifts and high-frequency noise. Channels contaminated by persistent noise were visually identified and excluded. The data were then cleaned using artifact subspace reconstruction to remove transient, high-amplitude artifacts, and previously excluded channels were interpolated using spherical interpolation. An independent component analysis (ICA) was performed to isolate and remove artifacts. Components associated with ocular, muscular, and cardiac activity were identified using a combination of automatic classification and visual inspection and subsequently removed. The data were then visually reviewed, and residual noisy segments were excluded from further analysis. Preprocessing procedures were consistently applied to both morning and evening recordings to ensure methodological uniformity across sessions.

For each EEG channel and for both morning and evening resting-state sessions, the power spectral density (PSD) was estimated via Welch’s method (512-sample Hamming window, 50% overlap, NFFT = 512) at a sampling rate of 250 Hz. From each PSD, absolute power was then computed by averaging all PSD values whose frequencies fell within 4–8 Hz.

### Statistical analysis, modeling, and correlation assessments

Statistical analyses and modelling were conducted to evaluate differences across the four study groups in demographics, clinical, actigraphy, and hd-EEG-derived measures. Group comparisons were performed for demographic and clinical variables, as well as for averaged values extracted from weekly actigraphy recordings. The assumption of normality for each variable was assessed using the Shapiro-Wilk test, and the homogeneity of variances across groups was verified using Levene’s test. The appropriate statistical tests were selected. When variables followed a normal distribution and exhibited homogeneity of variance, standard one-way ANOVA was applied, followed by Tukey’s HSD test for post hoc comparisons. For variables violating the assumption of normality but with homogeneous variance, the Kruskal-Wallis test was employed, followed by Dunn’s test for post hoc pairwise comparisons. In cases where both normality and homogeneity assumptions were not met, Welch’s ANOVA was used, followed by Games-Howell post hoc tests.

EEG data were primarily analyzed with a focus on theta power, extracted separately for the morning and evening resting-state sessions. To investigate between-group differences, specifically during the morning session, multiple linear regression (MLR) models were applied, incorporating *Group* as the main predictor:

1 $${{theta}}_{{MORNING}} \sim {Group}+{Age}+{Sex}$$

To evaluate within-group differences between morning and evening sessions, linear mixed-effects models (LMMs) were employed, with subject included as a random intercept to account for inter-individual variability, and *Group x Session* as the main predictor:

2 $${{theta}}_{\frac{{EVENING}}{{MORNING}}} \sim {Group}\times {Session}+{Age}+{Sex}+\left(1|ID\right)$$

All models were adjusted for potential confounding variables, including age and gender (LEDD and disease duration not included in the models, as both act as mediators rather than simple covariates). For spatial analyses based on topographical distributions, region-specific values were compared across sessions and groups using both MLR and LMM frameworks. To control for multiple comparisons across EEG channels, a non-parametric permutation approach (*n* = 2000) combined with Threshold-Free Cluster Enhancement (TFCE) was applied. This method tests the null hypothesis of exchangeable data labels and identifies statistically significant clusters without assuming predefined spatial distributions. This procedure was applied uniformly across all models, whether testing main effects, session-related changes, or *Group x Session* interactions, using the appropriate test statistic. For each contrast comparing the control group (CTL) with the clinical subgroups (EPD, ADV, DYS), null distributions were generated by shuffling group and session labels across subjects, enabling robust correction for spatial multiple comparisons^58^. A significance threshold of *p* < 0.05 was applied to all statistical tests. To complement these findings, effect sizes were also computed to quantify the magnitude of observed effects.

To evaluate hypothesis-driven relationships between theta power and clinical or sleep variables, we focused on logarithmically transformed theta measures extracted from the frontal region. This choice was motivated by previous EEG work in PD showing that low-frequency abnormalities in the 4–6 Hz range are most pronounced over centro-frontal electrodes, consistent with a frontal low-frequency dysrhythmia. Within this region of interest, we derived mean frontal theta values for each participant and session. For both morning theta power and the diurnal change in theta (evening minus morning), we first fitted linear models with theta as the dependent variable and age and sex as predictors. The residuals from these models were inspected for approximate normality and then entered into Pearson partial correlation analyses. Within each PD subgroup (EPD, ADV, DYS), we examined associations between frontal morning theta and LEDD, and between frontal diurnal theta change and LEDD. We treated the six subgroup correlations (three groups × two theta outcomes: morning and evening minus morning) as a single family of tests. To control the false discovery rate within this family, we applied the Benjamini–Hochberg false discovery rate (BH-FDR) correction to the corresponding *p* values and report both uncorrected *p* values and BH-FDR-adjusted *p* values. In the DYS group, we additionally explored the relationship between frontal theta measures and dyskinesia severity, indexed by the UDysRS Part IV. Finally, across all PD patients, we assessed correlations of frontal morning theta and diurnal theta change with estimated sleep metrics, mainly eSE and eWASO, applying the same BH–FDR correction to these correlations. For all correlation analyses, statistical significance was determined using a threshold of *p* < 0.05 for uncorrected p-values (e.g., UDysRS Part IV correlations) and pFDR < 0.05 on the BH–FDR-adjusted. The strength and direction of each association were quantified by the correlation coefficient ρ with 95% confidence intervals estimated using Fisher's z-transformation.

All procedures were conducted in accordance with the Declaration of Helsinki and were approved by the local ethics committee (Cantonal Ethical Commission, reference number CE4091). Written informed consent was obtained from all participants before study enrollment.

## Supplementary information

Supplementary information

## Data Availability

The datasets generated and/or analysed during the current study are not publicly available to protect patient confidentiality, but are available from the corresponding author on reasonable request.

## Code availability

The custom code used for data processing and analysis in this study is openly available at the following GitHub repository: https://github.com/biomedical-signal-processing/sleep-homeostasis-dyskinesia.
