# Supplementary material for: Altered wakeful theta activity characterizes levodopa-induced dyskinesia in Parkinson’s disease
Source: NPJ Parkinsons Dis. 2026 Mar 19;12:115. doi: 10.1038/s41531-026-01320-z (PMC13168226; doi:10.1038/s41531-026-01320-z)

Supplementary Tables

Supplementary Table S1. Region-wise summary of group comparisons for morning theta power. The table reports p values and Cohen's d effect sizes for group comparisons across the defined regions of interest (prefrontal, SMA, parietal, temporal, occipital) for morning theta power.

Region of interest	CTL vs EPD		CTL vs ADV		CTL vs DYS		EPD vs ADV		EPD vs DYS		ADV vs DYS	
**Brain Area**	*p*	*d*	*p*	*d*	*p*	*d*	*p*	*d*	*p*	*d*	*p*	*d*
Prefrontal	0.066	0.959	**0.006**	1.072	**0.007**	1.441	0.615	0.086	**0.050**	0.721	0.273	0.676
SMA	0.121	0.765	**0.005**	1.059	**0.006**	1.492	0.938	0.319	**0.028**	0.853	0.361	0.553
Parietal	0.078	0.953	**0.011**	1.116	**0.005**	1.66	0.804	0.223	**0.021**	0.900	0.291	0.715
Temporal	0.217	0.768	**0.013**	1.07	**0.022**	1.351	0.779	0.265	**0.051**	0.881	0.628	0.579
Occipital	0.131	0.890	**0.016**	0.957	**0.006**	1.545	0.738	0.122	**0.048**	0.704	0.521	0.545

ADV = advanced patients; CTL= control; DYS = dyskinetic patients; EPD= early-stage patients; SMA = Supplementary Motor Area.
p = p-value; Cohen's d = Effect size

Supplementary Table S2. Region-wise summary of group comparisons for diurnal theta power. The table reports p values and Cohen's d effect sizes for group comparisons across the defined regions of interest (prefrontal, SMA, parietal, temporal, occipital) for diurnal theta power changes (evening minus morning).

Region of interest	CTL vs EPD		CTL vs ADV		CTL vs DYS		EPD vs ADV		EPD vs DYS		ADV vs DYS	
**Brain Area**	*p*	*d*	*p*	*d*	*p*	*d*	*p*	*d*	*p*	*d*	*p*	*d*
Prefrontal	0.182	-0.559	0.186	-0.441	**0.005**	-1.623	0.295	0.345	0.444	-0.122	0.110	-0.891
SMA	0.131	-0.716	0.055	-0.941	**0.010**	-1.412	0.358	0.205	0.431	-0.149	0.281	-0.351
Parietal	0.136	-0.701	0.061	-0.861	**0.003**	-1.381	0.315	0.325	0.441	0.147	0.171	-0.516
Temporal	0.071	-1.181	0.191	-0.681	**0.050**	-0.981	0.181	0.781	0.333	0.363	0.223	-0.531
Occipital	0.088	-0.920	0.077	-0.701	**0.021**	-1.128	0.401	0.146	0.363	0.218	0.351	-0.210

ADV = advanced patients; CTL= control; DYS = dyskinetic patients; EPD= early-stage patients; SMA = Supplementary Motor Area.
p = p-value; Cohen's d = Effect size

Supplementary Figures

Supplementary Figure S1. Morning theta power differences across groups (unadjusted).
Group-level topographical maps of t-values from MLR models of log-transformed morning theta power (4-8 Hz). Maps show the direction and magnitude of group differences when contrasting each group with (A) CTL, (B) EPD, (C) ADV, and (D) DYS as the baseline (warm colors: higher theta than baseline; cool colors: lower theta than baseline). White dots mark electrodes with TFCE-corrected significant effects ($p < 0.05$). ADV = advanced patients without dyskinesia; CTL = controls; DYS = advanced patients with dyskinesia; EPD = early-stage patients.

Supplementary Figure S2. Diurnal theta power differences across groups (unadjusted).
Group-level topographical maps of t-values from LMMs of diurnal theta change (evening minus morning, 4-8 Hz). Maps show the direction and magnitude of group differences when contrasting each group with (A) CTL, (B) EPD, (C) ADV, and (D) DYS as the baseline (warm colors: greater diurnal increase than baseline; cool colors: reduced diurnal increase than baseline). White dots indicate electrodes with TFCE-corrected significant effects ($p < 0.05$). ADV = advanced patients without dyskinesia; CTL = controls; DYS = advanced patients with dyskinesia; EPD = early-stage patients.

Supplementary Figure S3. Correlation between theta power and sleep efficiency.
Scatterplots illustrate the correlations between residual log-transformed theta power measures (top row: morning theta; bottom row: diurnal theta change, defined as evening minus morning) and sleep efficiency, all adjusted for age and gender, in early-stage (EPD), advanced (ADV), and dyskinetic (DYS) patients. The six subgroup correlations (three groups × two theta outcomes: morning and evening minus morning) were treated as a single family of tests, and Benjamini–Hochberg false discovery rate (BH–FDR) correction was applied; both uncorrected p values and FDR-adjusted p values are reported in the panel labels. Each plot displays the best-fit regression line and shaded area representing the 95% confidence interval. ADV = advanced patients without dyskinesia; CTL = controls; DYS = advanced patients with dyskinesia; EPD = early-stage patients; SE = sleep efficiency.

Supplementary Figure S4. Correlation between theta power and wake after sleep onset.
Scatterplots illustrate the correlations between residual log-transformed theta power measures (top row: morning theta; bottom row: diurnal theta change, defined as evening minus morning) and wake after sleep onset, all adjusted for age and gender, in early-stage (EPD), advanced (ADV), and dyskinetic (DYS) patients. The six subgroup correlations (three groups × two theta outcomes: morning and evening minus morning) were treated as a single family of tests, and Benjamini–Hochberg false discovery rate (BH–FDR) correction was applied; both uncorrected p values and FDR-adjusted p values are reported in the panel labels. Each plot displays the best-fit regression line and shaded area representing the 95% confidence interval. ADV = advanced patients without dyskinesia; CTL = controls; DYS = advanced patients with dyskinesia; EPD = early-stage patients; WASO = wake after sleep onset.

Supplementary Figure S1. Morning theta power differences across groups (unadjusted).

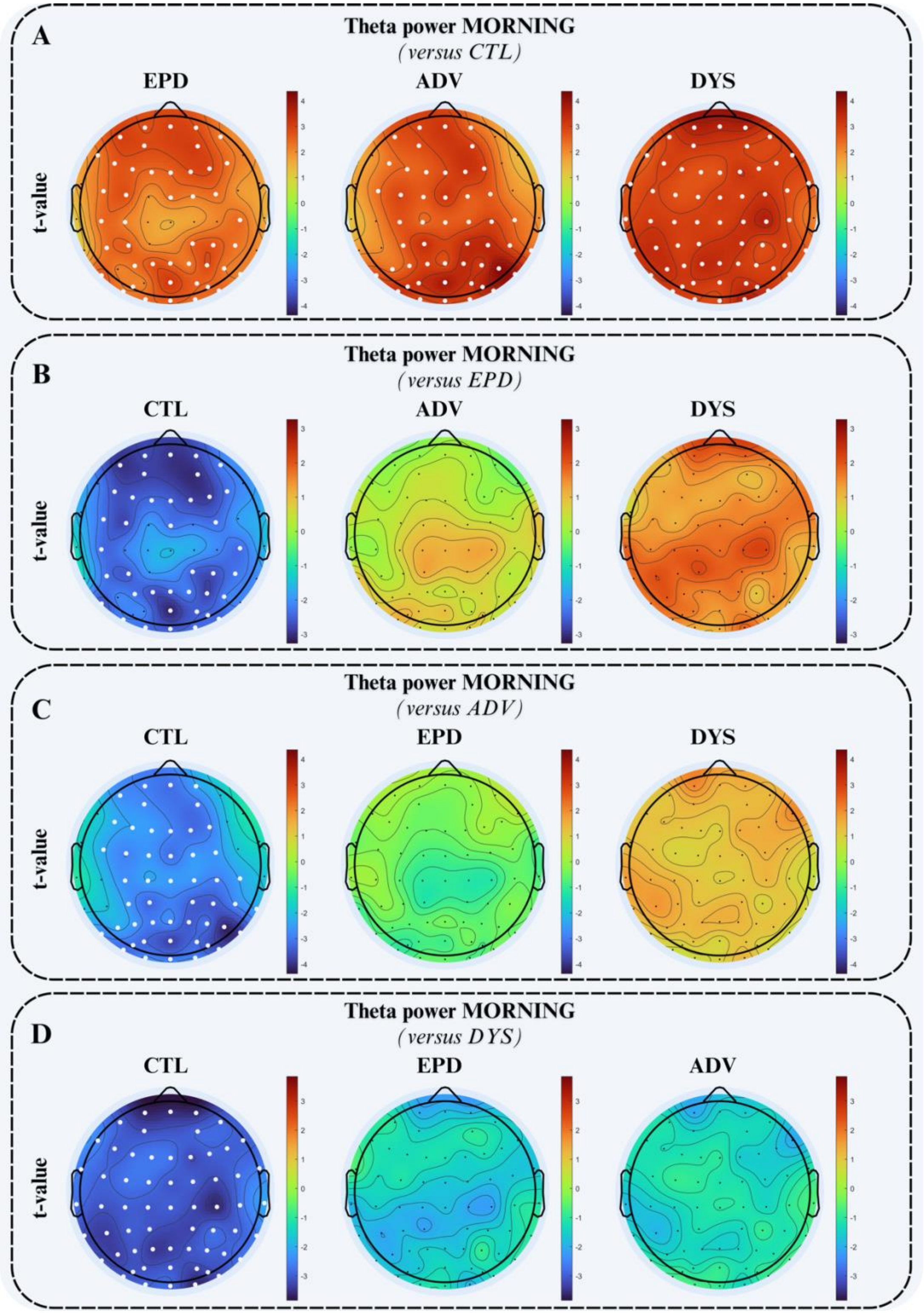

Supplementary Figure S2. Diurnal theta power differences across groups (unadjusted).

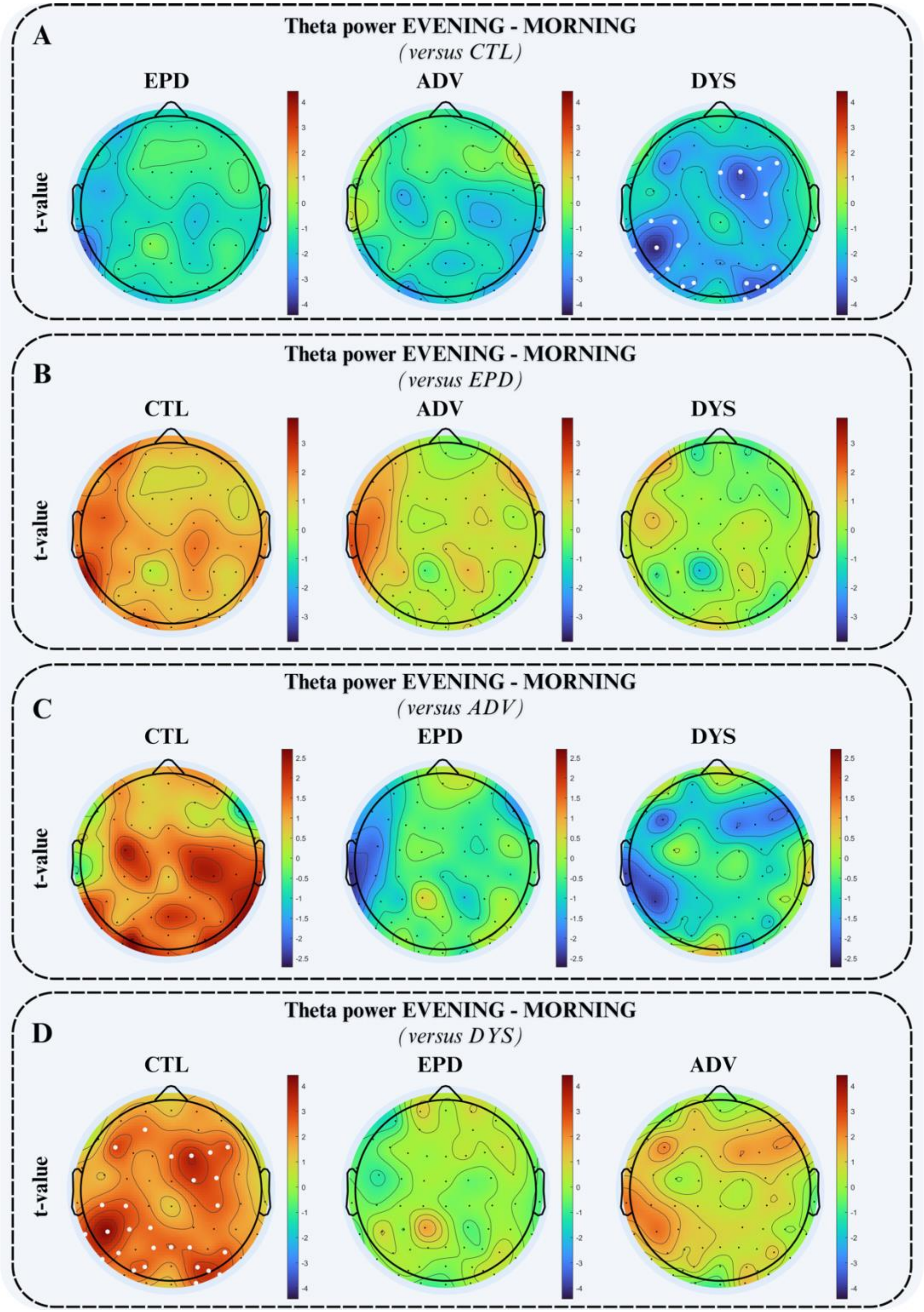

Supplementary Figure S3. Correlation between theta power and sleep efficiency.

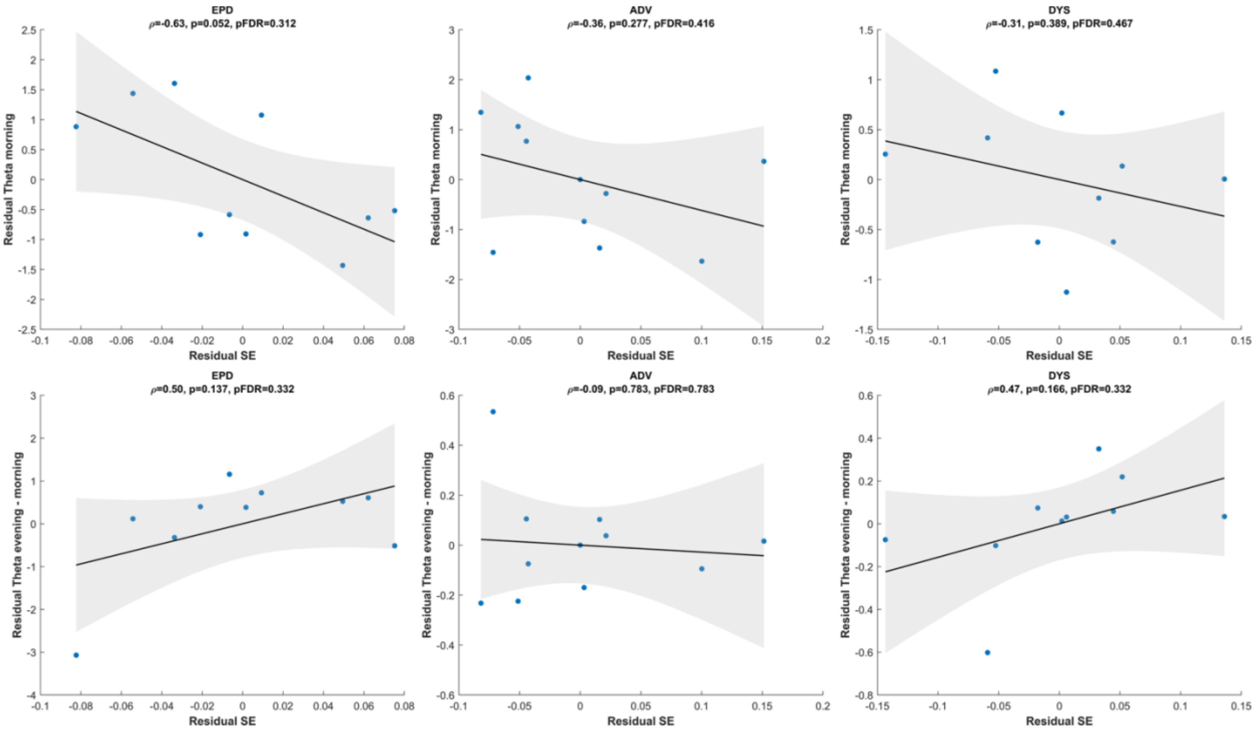

Supplementary Figure S4. Correlation between theta power and wake after sleep onset.

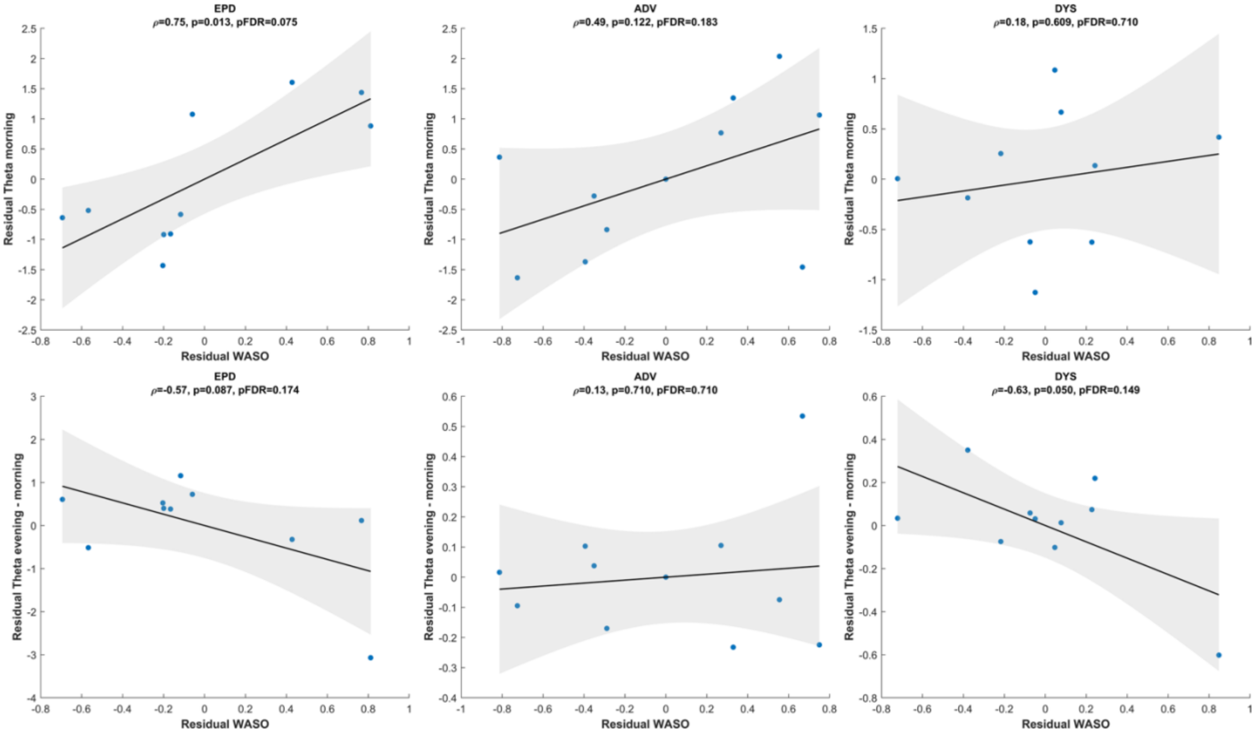

Supplement: Supplementary file 1 — Supplementary information [file 41531_2026_1320_MOESM1_ESM.pdf]
